# Promoting the therapeutic potential of interleukin-7 (IL-7) by expression in viral vectors

**DOI:** 10.1038/s41417-025-00960-2

**Published:** 2025-09-16

**Authors:** Myla Hudson, Robert H. Newman, Checo J. Rorie, Bryan L. Holloman, Howard L. Kaufman, Samuel D. Rabkin, Joseph Graves, Dipongkor Saha

**Affiliations:** 1 Department of Biology, College of Science and Technology, North Carolina Agricultural and Technical State University, Greensboro, NC USA; 2 Centers of Excellence in Biotechnology, North Carolina Agricultural and Technical State University, Greensboro, NC USA; 3 Department of Surgery, Massachusetts General Hospital and Harvard Medical School, Boston, MA USA; 4 Brain Tumor Research Center, Department of Neurosurgery, Massachusetts General Hospital and Harvard Medical School, Boston, MA USA

**Keywords:** Tumour immunology, Cancer immunotherapy

## Abstract

Interleukin 7 (IL-7) is an immunostimulatory cytokine essential for T cell development, proliferation, and maintenance. While IL-7 generates antitumor immunity, systemic IL-7 has not consistently produced strong anticancer effects. Achieving therapeutic cytokine concentrations in tumors often requires high systemic doses, leading to toxicity. To address this, localized cytokine expression within the tumor microenvironment (TME) has gained interest. One such approach involves cytokine expression by oncolytic viruses (OVs) that selectively replicate in cancerous cells while sparing ‘normal’ cells. Additionally, non-replicative viral vectors have become valuable tools for sustaining cytokine expression in the TME, inducing antitumor effects through non-lytic mechanisms. To effectively harness IL-7’s antitumor potential, both oncolytic and non-lytic viruses have been engineered to express IL-7, either alone or in combination with other immunomodulators, such as IL-12, IL-15, B7-1, or CCL19. Despite promising advancements, no comprehensive review exists on IL-7 expression in virus-based immunotherapy for cancer. Therefore, this manuscript aims to (i) summarize studies on viral IL-7 expression alone or with other immunomodulators, (ii) discuss the associated immune mechanisms of action, and (iii) explore opportunities for co-expressing IL-7 with other key cytokines to optimize immunovirotherapy strategies for cancer.

## Introduction

**Fig. 1:**
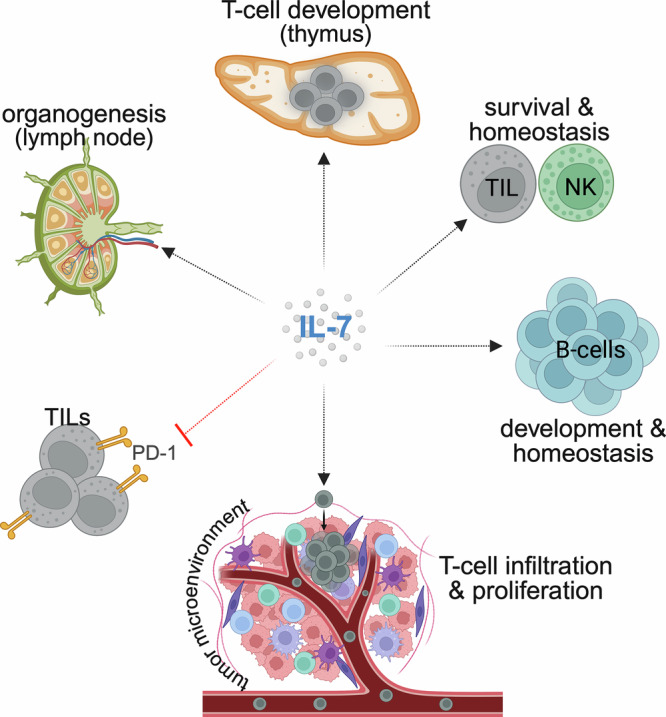
The general properties of IL-7 cytokine. IL-7 helps in organogenesis (e.g., lymph nodes), T-cell development centrally in thymus, survival and homeostasis of natural killer (NK) and tumor-infiltrating T cells (TILs), and development, maturation, and homeostasis of B lymphocytes. IL-7 also enhances infiltration and proliferation of cytotoxic T cells into the tumor microenvironment and prevents T-cell exhaustion by reducing programmed death 1 (PD-1) expression on TILs.

Interleukin 7 (IL-7), a common gamma (γ) chain cytokine family, is an immunomodulatory cytokine naturally produced by stromal cells in the bone marrow and epithelial cells in the thymus [1, 2]. IL-7 binds to a heterodimeric receptor, which is formed by the IL-7 receptor alpha chain (IL-7Rα) and the common γ chain [3]. Furthermore, IL-7Rα pairs with the thymic stromal lymphopoietin receptor (TSLPR) and forms another heterodimeric receptor for TSLP [1]. IL-7 interaction with the IL-7Rα subunit is essential for the activation of downstream signaling processes, such as phosphorylation- and redox-dependent signaling pathways, that impact cellular function in diverse ways [4]. For instance, IL-7Rα activation promotes phosphorylation of tyrosine residues on its intracellular domain, leading to the activation of kinases, such as Janus-associated kinase 1 (JAK1) or JAK3, that trigger additional signaling pathways involving Src, signal transducer and activator of transcription 5a/b (STAT5a/b), NADPH oxidase (NOX), and the phosphoinositide-3-kinase (PI3K)/mammalian target of rapamycin complex (mTORc)/Akt signaling axis [5]. In this way, IL-7 contributes to: (i) the development of T cells centrally in the thymus [6]; (ii) the long-term survival and homeostasis of natural killer (NK) cells and naïve, memory, and tumor-infiltrating T cells (TILs) in peripheral tissues [7]; (iii) B cell development, maturation, and homeostasis [7]; (iv) the proliferation of T cells, including cytotoxic T cells, and their infiltration into the tumor microenvironment (TME) [6]; (v) the prevention of immune exhaustion by reducing the expression of immune checkpoint molecules (e.g., PD-1) on TILs [8]; and (vi) the organogenesis of vital immune organs, such as lymph nodes [9] (Fig. 1). Due to these diverse properties, particularly its role in T-cell proliferation and homeostasis, IL-7 is considered a promising proinflammatory antitumor cytokine [5, 6]. However, IL-7 can be pro-tumorigenic, as it promotes cancer cell invasiveness by enhancing epithelial-mesenchymal transition [10]. Despite this duality, IL-7 is widely considered a beneficial T-cell immunomodulator [5, 6].

The antitumor efficacy of IL-7 as a native macromolecule has been extensively evaluated in preclinical models, demonstrating antitumor immunity [5]. Importantly, recombinant IL-7 has undergone clinical testing alone or alongside other immunotherapies to stimulate the antitumor activity of T cells in refractory cancers (NCT05075603, NCT04588038, NCT04710043) [11]. Additionally, IL-7 has been used as an ‘immune reconstitution’ agent to replenish T cells in immune-depleted cancer patients [11, 12]. However, systemic cytokine therapy as a native macromolecule, including high doses of IL-7, can cause adverse effects and toxicities [11, 13]. Thus, alternative approaches are required to harness the antitumor potential of IL-7.

Oncolytic viruses (OVs) are first-in-class immunotherapy agents that selectively replicate in cancerous cells, sparing ‘normal’ cells and inducing antitumor immunity (i.e., in situ vaccine effect) [14–17]. The effect of OV-induced anticancer vaccines can be further enhanced by engineering viruses to express immunostimulatory cytokines locally within the TME [18–24]. Among OVs, oncolytic herpes simplex virus (oHSV) is the most clinically advanced and the only OV approved by the U.S. Food and Drug Administration (FDA) for cancer treatment [25]. Non-replicative viral vectors have also emerged as promising tools in cancer immunotherapy [26]. Although they lack the oncolytic potential of OVs, they offer the advantage of sustained expression of immunostimulatory cytokines in the TME, thereby inducing antitumor effects through non-oncolytic (i.e., non-lytic) mechanisms [14, 26].

To safely utilize the antitumor potential of IL-7, lytic and non-lytic viral vectors have been engineered to express IL-7 locally within the TME [27–29]. Multiple studies have also explored the co-expression of IL-7 with other cytokines to achieve superior antitumor immunity. Examples include non-lytic Newcastle disease viruses (NDV) and oncolytic adenovirus, vaccinia, and herpes simplex viruses engineered to co-express IL-7 with IL-12 [30], IL-15 [31], B7-1 (CD86) [32], or CCL19 [33]. However, to date, no comprehensive review has compiled evidence related to IL-7 expression in the context of cancer immunovirotherapy. Thus, in this review, we aim to summarize studies involving viral expression of IL-7, either alone or in conjunction with other immunomodulators, and the associated immune responses and mechanisms of action.

## Viral expression of IL-7 as a single cytokine

### Viral expression of IL-7 induces tumor regression via T-cell activation

To date, three cytokines, IL-2, interferon alpha (IFNα), and an IL-15 receptor agonist, have been approved for cancer treatment [34, 35]. However, systemic cytokine therapy faces significant limitations, primarily due to poor cytokine accumulation in tumors following systemic delivery [13]. Achieving therapeutic concentrations in tumors often requires high-dose systemic cytokine therapy, which is associated with severe adverse effects before an optimal therapeutic concentration can be achieved [13]. To address these challenges, Kudling et al. engineered an oncolytic adenovirus (oAd) expressing human IL-7, designated Ad5/3-E2F-d24-hIL7 (TILT-517), for localized delivery of IL-7 directly into tumors [27]. Intratumoral administration of TILT-517 in an immunocompetent subcutaneous HapT1 pancreatic cancer model in Syrian hamsters significantly controlled tumor burden compared to the Ad5/3-E2F-d24 control lacking IL-7 expression [27].

**Fig. 2:**
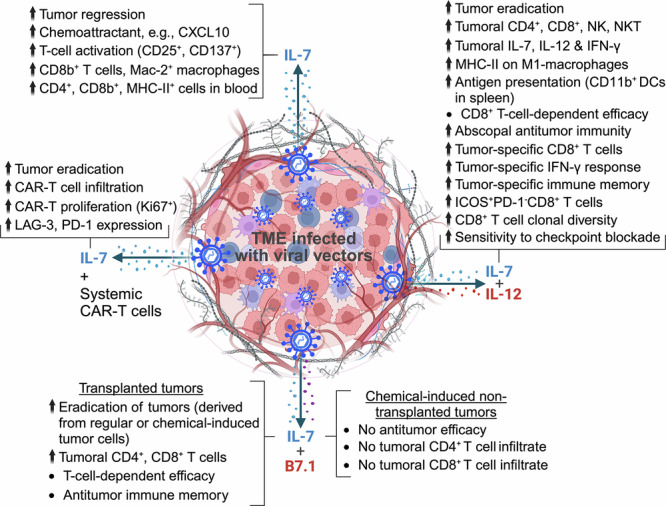
Diverse (antitumor) effects of intratumorally injected viral vectors expressing IL-7 or co-expressing IL-7 with IL-12 or B7.1. An upward arrow indicates ‘enhanced.’ TME, tumor microenvironment; LAG-3, lymphocyte activation gene 3; PD-1, programmed death 1.

Human IL-7 expression (by the TILT-517) shows cross-reactivity in hamsters, leading to antitumor activity by the TILT-517 in a Syrian hamster model [27]. This was associated with significant upregulation of T cell activation markers, such as CD25 and CD137, within tumors, increased tumoral infiltration of CD8^+^ T cells and Mac-2^+^ monocytes/macrophages, and elevated levels of CD4^+^, CD8^+^ T, and MHCII^+^ cells in the blood (Fig. 2; Table 1). Similarly, in patient-derived xenograft models of ovarian cancer receiving an intraperitoneal infusion of peripheral blood mononuclear cells, intratumoral TILT-517 resulted in significant tumor inhibition compared to a non-IL7-expressing oAd [27].

**Table 1:** IL-7-based viral vectors in cancer therapy.

Name	Description	Tested model(s)	Treatment route	Major outcomes	Refs.
Ad5/3-E2F-d24-hIL7 (TILT-517)	Oncolytic adenovirus (oAd) coding human IL-7	HapT1 pancreatic cancer	Intratumoral	○ Significantly controlled tumor burden compared to control oAd (Ad5/3-E2F-d24) ○ Increased T cell activation and intratumoral infiltration of CD8^+^ T cells and Mac-2^+^ monocytes/macrophages ○ Elevated levels of CD4^+^, CD8^+^ T, and MHCII^+^ cells in the blood	[27]
		Patient-derived xenograft model of ovarian cancer	Intratumoral	○ Significantly inhibited tumor growth compared to a non-IL7-expressing oAd in mice receiving an intraperitoneal infusion of peripheral blood mononuclear cells	[27]
		Ex vivo patient-derived ovarian cancer samples	Direct inoculation	○ Induced a higher pro-inflammatory/anti-inflammatory cytokine ratio than Ad5/3-E2F-d24 or mock ○ Substantial increase in ex vivo recruitment of cytotoxic CD4^+^ and CD8^+^ T cells in TILT-517-treated samples	[27]
LX/IL-7	A non-lytic Newcastle disease virus (NDV) expressing IL-7	B16-F10; EL-4	Subcutaneous injections with B16-LX/IL-7 or EL4-LX/IL7 [B16-LX/IL-7 or EL4-LX/IL7: LX/IL-7 loaded in irradiated B16-F10 murine melanoma (i.e., B16-LX/IL-7) or EL-4 murine lymphoma cells (i.e., EL4-LX/IL7)]	Prophylactic: ○ B16-LX/IL-7 or EL4-LX/IL7 significantly inhibited homologous but not heterologous tumor growth compared to vaccination with irradiated B16-F10 cells loaded with LX strain expressing a red fluorescent protein (RFP) ○ Enhanced tumor-specific IFN-γ response Therapeutic: ○ B16-LX/IL-7 or EL4-LX/IL7 significantly inhibited homologous tumor growth (vs. controls) ○ Significantly increased infiltration of both CD4^+^ and CD8^+^ T cells into tumors (vs. controls), with an enhanced tumor-specific IFN-γ response ○ CD8^+^ T cell-dependent efficacy	[28]
oAd-IL7	oAd-expressing human IL-7	Orthotopic glioblastoma xenograft	Intratumoral oAd-IL7 plus intravenous B7-H3-expressing CAR-T cells	○ The combination resulted in a significant proportion of long-term survivors (i.e., 80%) ○ Increased tumoral infiltration of B7H3-CAR-T cells and significantly higher levels of Ki67 in the infiltrated CAR-T cells ○ Increased expression of T-cell exhaustion markers, such as LAG-3 and PD-1	[29]
hIL7-VV, mIL12-VV	Oncolytic vaccinia virus expressing human IL-7 or murine IL-12	LLC lung carcinoma	Intratumoral	○ IL-7 expression (hIL7-VV) had no significant antitumor effects ○ IL-12 expression (mIL12-VV) inhibited tumor growth, achieving a complete response in 1/7 (14.3%) animals ○ The combination of hIL7-VV plus mIL12-VV (versus monotherapies) resulted in 57.1% (4/7) complete response rate, which was associated with a significant intratumoral infiltration of CD4^+^ T and NKT cells	[30]
hIL7/mIL12-VV	Oncolytic vaccinia virus co-expressing human IL-7 plus murine IL-12	B16-F10; CT26.WT	Intratumoral	○ Viral co-expression of IL-7 plus IL-12 (hIL7/mIL12-VV), resulted in a complete response in 75% (6/8) animals compared to 25% (2/8) in the cont-VV control group in B16-F10 model ○ CD8^+^ (not CD4^+^) T cell-dependent efficacy in the CT26.WT model	[30]
		Bilateral CT26.WT	Intratumoral	○ hIL7/mIL12-VV injection into one tumor resulted in 100% (6/6) eradication of injected tumors and 50% (3/6) of non-injected contralateral tumors ○ Viral DNA was detected only in injected tumors ○ Significantly increased intratumoral infiltration of conventional T (CD4^+^FoxP3^-^), CD8^+^ T, NKT, NK, and regulatory T cells (CD4^+^FoxP3^+^) in both injected and distant tumors ○ Generated tumor antigen-specific (gp70^+^CD8^+^) T cells in both injected and distant tumors ○ Development of tumor-specific immune memory ○ Synergizes with anti-PD-1 or anti-CTLA-4 and eradicated 60% and 40% of non-injected tumors	^(30)^
		Bilateral LLC	Intratumoral	○ hIL7/mIL12-VV reduced tumor growth by 43.1% in both injected and non-injected tumors ○ Viral DNA was present in both tumors	[30]
hIL7/hIL12-VV	Oncolytic vaccinia virus co-expressing human IL-7 and human IL-12	HCT 116; U87; Detroit 562	Intratumoral	○ hIL7/hIL12-VV led to significant tumor regression compared to mock treatment in all three subcutaneous tumor models in nude mice	[30]
		NCI-H1373	Intratumoral	○ hIL7/hIL12-VV was significantly more effective than cont-VV in reducing tumor burden in humanized mice bearing subcutaneous NCI-H1373 tumors ○ Enhanced intratumoral infiltration of CD4^+^, CD8^+^ T, NKT, and NK cells compared to cont-VV or PBS	[30]
SINV-IL7 or SINV-IL12	Sindbis virus (SINV) expressing IL-7 or IL-12	U-87MG GBM	Intratumoral	○ SINV-IL7 or SINV-IL12 demonstrated superior efficacy compared to control SINV in subcutaneously implanted U-87MG GBM model	[61]
SINV-IL7/IL12	A SINV co-expressing IL-7 plus IL-12	Intracranial U-87MG GBM	Intratumoral	○ SINV-IL7/IL12 enhanced tumor suppression compared to SINV-IL7 or SINV-IL12, with SINV-IL7/IL12 achieved 80% long-term survivors	[61]
LX/IL(15 + 7)	A non-lytic NDV LX strain co-expressing IL-7 plus IL-15	B16-F10; EL-4	Subcutaneous injections with B16-LX/IL(15 + 7) [B16-LX/IL(15 + 7) is described as irradiated B16-F10 cells loaded with LX/IL(15 + 7) virus]	Prophylactic: ○ LX/IL(15 + 7)-modified B16-F10 cells (B16-LX/IL(15 + 7)) or control LX/RFP-modified B16-F10 cells (i.e., B16-LX/RFP) significantly inhibited B16-F10 tumor growth compared to irradiated B16-F10 cells without virus loading ○ The B16-LX/IL(15 + 7) vaccine demonstrated a significantly superior prophylactic antitumor effect than the control B16-LX/RFP vaccine ○ Both B16-LX/IL(15 + 7) and B16-LX/RFP vaccines similarly enhanced infiltration of CD4^+^ and CD8^+^ T cells into tumors compared to irradiated B16-F10 cells Therapeutic: ○ The therapeutic B16-LX/IL(15 + 7) vaccine significantly inhibited B16-F10 tumor growth compared to the B16-LX/RFP vaccine ○ The antitumor effect of the B16-LX/IL(15 + 7) vaccine was tumor-specific ○ B16-LX/IL(15 + 7) vaccine significantly increased intratumoral infiltration of CD3^+^, CD4^+^, and CD8^+^ T cells (vs. B16-LX/RFP), and the vaccine efficacy was CD8^+^ T cell-dependent	[31]
Ad.IL-7/B7.1	A replication-defective adenovirus co-expressing IL-7 plus B7.1	TS/A adenocarcinoma	Intratumoral	○ Ad.IL-7/B7.1 treatment led to 70% (7/10) tumor-free long-term survivors compared to no survivors in the Ad.βgal group in transplanted TS/A model ○ Ad.IL-7/B7.1 treatment enhanced the infiltration of CD4^+^ and CD8^+^ T cells into transplanted TS/A tumors compared to the control, and its efficacy was abrogated in the absence of T cells ○ Ad.IL-7/B7.1 treatment provided 100% immune memory protection	[32]
oHSV2-IL7×CCL19	A type 2 oHSV co-expressing IL-7 plus CCL19	CT26	Intratumoral	○ The oHSV2-IL7×CCL19 treatment showed better, but not statistically significant, efficacy than the other control viruses expressing IL-12, anti-PD-1, or IL-15	[82]

In ex vivo patient-derived ovarian cancer samples, TILT-517 infection significantly increased the level of pro-inflammatory cytokines while reducing anti-inflammatory cytokines, resulting in a higher pro-inflammatory/anti-inflammatory cytokine ratio than Ad5/3-E2F-d24 or mock. These findings suggest that the viral expression of IL-7 creates a pro-inflammatory TME. This concept was further validated when TILT-517-infected ovarian cancer samples showed significantly higher levels of chemoattractant (e.g., C-X-C motif chemokine ligand 10 (CXCL10)) compared to controls, leading to a substantial increase in ex vivo recruitment of cytotoxic CD4^+^ and CD8^+^ T cells in TILT-517-treated samples [27] (Fig. 2; Table 1).

Overall, IL-7 expression within tumors creates a pro-inflammatory TME, a key characteristic for successful immunotherapy involving an immune checkpoint inhibitor (ICI) [36]. Thus, further studies should explore the in-depth therapeutic potential of TILT-517 in combination with ICIs.

### Viral expression of IL-7 induces tumor-specific immunity and improves the efficacy of autologous vaccine

Zhao et al. developed a non-replicative IL-7-expressing NDV by inserting the IL-7 transgene into the genome of the LX strain, a non-lytic NDV. The modified virus was designated as LX/IL-7 [28]. Subsequently, they developed an LX/IL-7-based autologous tumor vaccine by loading irradiated B16-F10 murine melanoma or EL-4 murine lymphoma cells with LX/IL-7 (i.e., B16-LX/IL-7 or EL4-LX/IL7) and tested its antitumor efficacy, prophylactically and therapeutically [28].

**Fig. 3:**
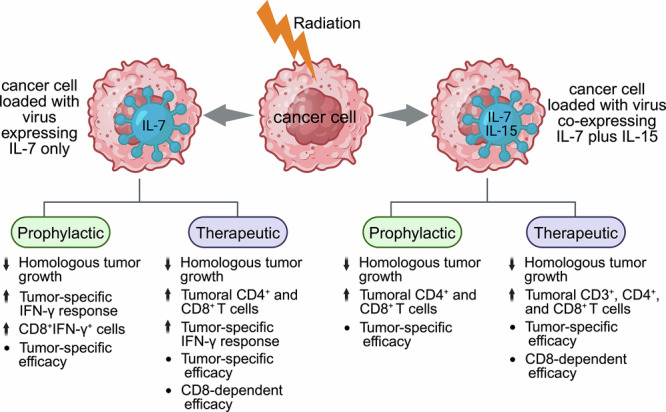
The prophylactic and therapeutic effects of irradiated autologous tumor cell vaccine loaded with viral vectors expressing IL-7 (left panel) or co-expressing IL-7 plus IL-15 (right panel). The upward and downward arrows indicate ‘enhanced’ and ‘reduced’ functions, respectively, of irradiated cancer cells loaded with viral vectors.

Prophylactically, subcutaneous immunization with B16-LX/IL-7 significantly inhibited homologous B16-F10 tumor growth compared to vaccination with irradiated B16-F10 cells loaded with LX strain expressing a red fluorescent protein (RFP) (i.e., B16-LX/RFP), highlighting the antitumor role of IL-7 expression. This finding was also similarly reproduced in the EL-4 model. The co-culture of splenocytes with B16 cell lysate demonstrated significantly more IFN-γ-expressing CD8^+^, not CD4^+^, T cells in the B16-LX/IL-7 group compared to controls [28]. This IFN-γ response was tumor-specific, as splenocytes harvested from B16-LX/IL-7-treated mice efficiently killed B16-F10 cells but not antigenically unrelated EL-4 cells. The tumor specificity of the B16-LX/IL-7 vaccine was further confirmed in vivo, where the B16-LX/IL-7 vaccine controlled B16-F10 tumor growth but failed to inhibit EL-4 lymphoma tumors [28] (Fig. 3; Table 1).

Therapeutically, the B16-LX/IL-7 vaccine significantly inhibits homologous B16-F10 tumor growth compared to the non-IL-7-expressing B16-LX/RFP vaccine. This was also reproduced in the EL-4 model using the autologous EL4-LX/IL-7 vaccine. The IL-7-expressing vaccine led to significantly increased infiltration of both CD4^+^ and CD8^+^ T cells into tumors (vs. controls), and the therapeutic efficacy of the B16-LX/IL-7 vaccine was CD8^+^ T cell-dependent [28]. The vaccine efficacy was also tumor-specific in the therapeutic settings since the EL4-LX/IL-7 vaccine did not work against antigenically unrelated B16-F10 tumors (Table 1). Furthermore, the B16-LX/IL-7 vaccine induced a tumor-specific IFN-γ response, as splenocytes or TILs from B16-LX/IL-7-treated mice failed to produce IFN-γ when stimulated with EL-4 tumor cell lysate [28] (Fig. 3).

In summary, an autologous tumor cell vaccine loaded with a non-lytic NDV expressing IL-7 (i.e., LX/IL-7) provides significant prophylactic and therapeutic benefits by activating tumor-specific cytotoxic T-cell responses. Autologous tumor cell vaccine loaded with NDV without IL-7 expression has shown promising results in clinical studies [37], but the superior antitumor efficacy of autologous vaccine loaded with IL-7-expressing NDV in preclinical studies suggests that IL-7 expression offers substantial therapeutic advantages. However, before this vaccine can be brought into clinical translation, further in vivo characterization is necessary to evaluate the kinetics of IL-7 and the potential toxicities associated with IL-7 release. While IL-7 expression alone does not achieve complete tumor eradication, it significantly enhances vaccine efficacy associated with cytotoxic T cell responses [28]. This suggests that T cell-based immunotherapies, such as ICIs, chimeric antigen receptor T cells (CAR-T), or bi-specific T cell engagers, may produce synergistic effects when combined with an autologous tumor cell vaccine loaded with IL-7-expressing NDV. However, as the LX strain of NDV is non-lytic and does not induce oncolysis for at least 24 h post-infection [28], the release of tumor antigens and/or IL-7 may be limited. Future studies should explore the use of a lytic NDV strain expressing IL-7 and evaluate its efficacy in parallel with the non-lytic NDV strain expressing IL-7, with or without being loaded into autologous tumor cells.

### Viral expression of IL-7 stimulates the proliferation of CAR-T cells and improves antitumor efficacy

B7-H3 (CD276) is an immune checkpoint molecule overexpressed in many cancer types, including 76% of glioblastoma (GBM) tumors [38], the most common primary malignant brain tumor in adults [39, 40]. B7-H3 is linked to tumor progression, therapy resistance, and cellular invasion [41]. Huang et al. constructed CAR-T cells targeting B7-H3 (referred to as B7H3-CAR-T) along with an oAd-expressing human IL-7 (oAd-IL7) and evaluated their combinatorial effect in orthotopic GBM models [29].

B7H3-CAR-T cells exposed to oAd-IL7-infected GBM cells demonstrated significantly greater proliferation and survival compared to B7H3-CAR-T cells exposed to control oAd-infected GBM cells [29]. This study underscores the beneficial role of IL-7 in enhancing T-cell survival and expansion. In vivo, in an orthotopic GBM xenograft model, the combination of oAd-IL7 and B7H3-CAR-T resulted in a significant proportion of long-term survivors (i.e., 80%), while the monotherapy using either oAd-IL7 or B7H3-CAR-T alone did not yield any survivors. The enhanced efficacy of the combinatorial therapy was associated with (i) increased tumoral infiltration of B7H3-CAR-T cells and (ii) significantly higher levels of Ki67 (a marker for proliferating cells) in the infiltrated B7H3-CAR-T cells. These effects were also accompanied by increased expression of T-cell exhaustion markers, such as LAG-3 and PD-1 [29] (Fig. 2; Table 1).

In summary, the IL-7 expression enhances the survival and expansion of CAR-T cells, resulting in promising antitumor efficacy and long-term survival. This combination strategy primarily targets B7-H3-expressing GBMs [38] but can also be applied to other B7-H3-expressing tumors, ensuring broader applicability. The safety of this compelling combination therapy needs to be assessed before clinical translation.

## Viral co-expression of IL-7 with IL-12

While the expression of single cytokines has provided important data on their contribution to therapeutic antitumor responses, in general, the narrow therapeutic window for many cytokines has precluded meaningful efficacy against cancers [20, 23, 42–46]. Similarly, the viral expression of IL-7 alone does not consistently eradicate cancers or achieve durable efficacy [27–29]. One strategy to improve efficacy is to consider the expression of more than one immunomodulator [14, 21, 22, 47, 48]. To enhance the antitumor immunity of localized IL-7 expression, several viruses have been engineered to co-express IL-7 alongside other immunostimulatory cytokines [30–33]. This section and the following sections will summarize studies involving viruses that co-express IL-7 with another cytokine, highlighting their antitumor potential and mechanisms of action.

### Viral co-expression of IL-7 plus IL-12 induces cytotoxic T cell-driven antitumor immunity

IL-12 activates dendritic cells (DCs) [49], stimulates macrophages and NK cells [18, 49], promotes T cell proliferation, and skews CD8^+^ T cells toward a cytotoxic phenotype [50, 51]. These diverse immunostimulatory properties make it a master proinflammatory cytokine, which has been tested clinically to treat cancer [52–55]. However, systemic IL-12 therapy causes severe toxicities, necessitating localized expression within the TME [56, 57]. Clinical studies with controlled release of IL-12 within the TME are ongoing [58].

Nakao et al. generated an oncolytic vaccinia virus co-expressing human IL-7 plus murine IL-12 (hIL7/mIL12-VV) and compared its efficacy with single cytokine-expressing (hIL7-VV, mIL12-VV) or non-cytokine-expressing (cont-VV) viruses in murine models [30]. Since human IL-7 is biologically active in mice [59], but human IL-12 is not [60], the study used murine IL-12. In the LLC lung carcinoma model, intratumoral injections of hIL7-VV had no significant antitumor effects, while IL-12 expression (mIL12-VV) inhibited tumor growth, achieving a complete response in 1/7 (14.3%) animals. The combinatorial use of hIL7-VV plus mIL12-VV further improved outcomes, with a 57.1% (4/7) complete response rate [30] (Fig. 2; Table 1). This enhanced efficacy correlated with increased intratumoral infiltration of CD4^+^ and CD8^+^ T, NK, and natural killer T (NKT) cells, with a significant effect observed on CD4^+^ T and NKT cells. Increased IFN-γ production in TME likely contributed to this effect (Fig. 2). Importantly, mice receiving the combinatorial treatment experienced no weight loss, suggesting that viral co-expression of dual cytokines within the tumor is well tolerated [30].

In the B16-F10 melanoma model, viral co-expression of IL-7 plus IL-12 (hIL7/mIL12-VV), instead of using two separate viruses (hIL7-VV plus mIL12-VV), resulted in a complete response in 75% (6/8) animals compared to 25% (2/8) in the cont-VV control group [30]. Dual cytokine expression by hIL7/mIL12-VV was also efficacious against poorly immunogenic TRAMP-C2 prostate tumors and advanced-stage (>160 mm^3^) CT26.WT tumors [30]. The antitumor activity of hIL7/mIL12-VV in the CT26.WT model was completely abrogated in the absence of CD8^+^ (not CD4^+^) T cells, suggesting a CD8^+^ T-cell-dependent mechanism [30]. However, due to the lack of relevant control viruses (i.e., hIL7-VV or mIL12-VV), it was unclear whether the observed efficacy was driven by the expression of one or both cytokines.

The antitumor role of the viral expression of IL-7 and/or IL-12 was further supported by another study [61]. For example, intratumoral injections of sindbis virus (SINV) expressing IL-7 or IL-12 (SINV-IL7 or SINV-IL12) in a subcutaneous U-87MG GBM model demonstrated superior efficacy compared to control SINV. A SINV co-expressing IL-7 plus IL-12 (SINV-IL7/IL12) further enhanced tumor suppression compared to SINV-IL7 or SINV-IL12, with SINV-IL7/IL12 achieved 80% long-term survivors in an intracranial U-87MG GBM model (Table 1). However, this study did not define the antitumor immune mechanisms of IL-7 and/or IL-12 expression [61].

### Viral co-expression of IL-7 plus IL-12 induces abscopal tumor-specific immunity and immunologic memory

A key aspect of successful immunovirotherapy is assessing whether virus-induced immunostimulation generates systemic antitumor immunity (i.e., abscopal response) [62]. The dual cytokine-expressing hIL7/mIL12-VV effectively controlled distant tumor growth in bilateral CT26.WT and LLC models [30]. In the CT26.WT model, intratumoral injection of hIL7/mIL12-VV into one tumor resulted in 100% (6/6) eradication of injected tumors and 50% (3/6) of non-injected contralateral tumors, whereas cont-VV failed in both (Table 1). The inhibition of distant tumor growth by hIL7/mIL12-VV suggests its potential to manage metastatic diseases [30]. Although hIL7/mIL12-VV controlled non-injected tumors, viral DNA was detected only in injected tumors [30], indicating virus-induced systemic immunity rather than a direct oncolytic effect [62]. This correlated with enhanced MHC-II expression on antitumoral M1-like macrophages in non-injected tumors and spleens and elevated CD11b^+^ DCs in spleens [30] (Fig. 2). This suggests that hIL7/mIL12-VV treatment facilitated robust antigen presentation in distant lymphoid organs (e.g., spleens), critical for antitumor immunity [14].

Consistently, compared to cont-VV, hIL7/mIL12-VV significantly increased intratumoral infiltration of conventional T (CD4^+^FoxP3^-^), CD8^+^ T, NKT, NK, and regulatory T cells (CD4^+^FoxP3^+^) in both injected and distant tumors. Interferon gamma (IFN-γ) levels were significantly elevated following hIL7/mIL12-VV treatment, likely contributing to increased PD-L1 expression [45, 63] in both injected and non-injected CT26.WT tumors [30]. Furthermore, hIL7/mIL12-VV generated gp70 tumor antigen-specific (gp70^+^CD8^+^) T cells in both injected and distant CT26.WT tumors (Fig. 2; Table 1), reinforcing its systemic antitumor immune effect [30].

In the bilateral LLC model, hIL7/mIL12-VV reduced tumor growth by 43.1% in both injected and non-injected tumors [30]. Antitumor effects in non-injected tumors indicate virus-induced abscopal immunity [62]. Unlike CT26.WT model, viral DNA was present in both tumors. Mice cured of CT26.WT tumors rejected tumor rechallenge and remained tumor-free, suggesting the development of immune memory (Table 1). This immune memory was tumor-specific, as IFN-γ secretion (by splenocytes harvested from mice cured of CT26.WT tumors) was significantly higher against CT26.WT cells than antigenically unrelated cancer cells [30] (Fig. 2).

### Viral co-expression of IL-7 plus IL-12 increases CD8^+^ T cell clonal diversity

The antitumor efficacy of hIL7/mIL12-VV was also evaluated in humanized tumor models. To achieve this, a new vaccinia virus co-expressing human IL-7 and human IL-12 (hIL7/hIL12-VV) was engineered, and its efficacy was assessed in immunocompromised mice bearing human HCT 116 colon tumors, U87 glioblastoma, or Detroit 562 head and neck tumors [30]. In all three models, hIL7/hIL12-VV led to significant tumor regression compared to mock treatment. Similarly, in humanized mice bearing subcutaneous NCI-H1373 tumors, hIL7/hIL12-VV was significantly more effective than cont-VV in reducing tumor burden. The superior efficacy of hIL7/hIL12-VV was associated with enhanced intratumoral infiltration of CD4^+^, CD8^+^ T, NKT, and NK cells compared to cont-VV or PBS [30] (Fig. 2; Table 1).

In a follow-up study, mechanisms of the hIL7/hIL12-VV virus were investigated and compared with corresponding controls, specifically involving viruses with or without IL-7 or IL-12 expression [64]. Interestingly, IL-7 expression alone did not promote the clonality of tumor-infiltrating CD8^+^ T cells. In contrast, IL-12 expression significantly enhanced CD8^+^ T cell clonality compared to IL-7 expression alone. The viral expression of IL-7 plus IL-12 within tumors increased the clonal diversity of CD8^+^ T cells compared to IL-7 expression; however, this combinatorial effect was not statistically different from IL-12 expression [64]. In another study, the same group demonstrated that viral co-expression of IL-7 and IL-12 by hIL7/mIL12-VV, compared to cont-VV treatment, significantly enhanced the percentage of ICOS^+^PD-1^-^CD8^+^ effector T cells within tumors in both CT26.WT and LLC models [65] (Fig. 2). Since inducible costimulatory (ICOS) is a marker for CD4^+^ helper T cells contributing to humoral immunity [66], this suggests that hIL7/mIL12-VV elicits humoral immunity in this context.

### Viral co-expression of IL-7 plus IL-12 synergizes with ICIs

Virotherapy can be combined with systemic treatment to achieve better therapeutic outcomes [67]. In the bilateral CT26.WT model, the antitumor efficacy of hIL7/mIL12-VV was evaluated in combination with anti-PD-1 or anti-CTLA-4. Virotherapy (i.e., hIL7/mIL12-VV) alone eradicated only 10% of non-injected contralateral tumors (which mimic metastasis), while neither anti-PD-1 nor anti-CTLA-4 monotherapy showed efficacy. Importantly, combining hIL7/mIL12-VV with anti-PD-1 or anti-CTLA-4 eradicated 60% and 40% of non-injected contralateral tumors, respectively, showing the combinatorial effect [30] (Fig. 2; Table 1).

Overall, although IL-7 expression alone was not significantly beneficial in the models discussed above, it did appear to enhance the immune response elicited by IL-12 expression, as demonstrated in the LLC model. The above studies indicate that the viral co-expression of IL-7 and IL-12 within tumors effectively modifies the immune status of the TME both locally and systemically, enabling previously non-responsive tumors (such as CT26.WT and LLC) to become responsive to ICI without compromising safety [30]. Further, the local induction of IFN-γ may drive PD-L1 expression, which can also sensitize tumors to ICI [45]. Thus, the use of oncolytic cytokine-encoded viruses represents an increasingly popular strategy for combination immunovirotherapy, exploiting cytokine-expressing viruses to convert tumors from an immunologically ‘cold’ state to an immunologically ‘hot’ one, thereby sensitizing them to ICI [45, 67, 68].

The viral co-expression of IL-7 and IL-12 in one tumor alters the immune status of non-injected tumors and significantly regresses non-injected tumors [30], suggesting a strong induction of whole-body antitumor immunity, essential for targeting metastatic diseases [69]. Importantly, all cured mice treated with the dual cytokine-expressing vaccinia virus developed long-term immune memory responses, crucial for preventing recurrence [70]. Likewise, a combinatorial study using tumor matrix (collagen)-binding IL-7 and IL-12 showed synergistic antitumor effects associated with the induction of immunologic memory [71]. However, a limitation of most of the studies discussed in this section was the lack of single cytokine-encoding viruses as controls, making it challenging to delineate the specific role of each cytokine. Nevertheless, the strong antitumor effects observed with the virus co-expressing IL-7 and IL-12 underscore its clinical translatability once the antitumor role of each individual cytokine (IL-7 or IL-12) is defined preclinically.

## Viral co-expression of IL-7 with IL-15

IL-7, a member of the common γ-chain cytokine family, maintains memory CD8^+^ T cell responses [6, 7, 72]. Like IL-7, IL-15 is another γ-chain cytokine maintaining memory CD8^+^ T cell responses [73]. Additionally, like IL-7, IL-15 promotes proliferation and activation of T and NK cells [74]. Because both IL-7 and IL-15 produce similar immunostimulatory effects on the host immune cells, especially T cells [75], it is believed that their co-expression by a virus can lead to superior antitumor immunity compared to single cytokine expression.

### Viral co-expression of IL-7 plus IL-15 induces tumor-specific prophylactic antitumor immunity

As described above, irradiated B16-F10 cells loaded with non-lytic NDV LX strain expressing IL-7 (i.e., B16-LX/IL-7) were utilized as an autologous tumor cell vaccine [28]. Xu et al. further modified the LX strain to co-express IL-7 and IL-15, separated by a 2A peptide derived from the foot-and-mouth disease virus, creating LX/IL(15 + 7) [31]. As an improvement on the B16-LX/IL-7 vaccine [28], irradiated B16-F10 cells (treated with 200 Gy of radiation) were loaded with LX/IL(15 + 7), here referred to as the B16-LX/IL(15 + 7) vaccine [31].

Subcutaneous prophylactic immunization of C57BL/6 mice with LX/IL(15 + 7)-modified B16-F10 cells (B16-LX/IL(15 + 7)) or LX/RFP-modified B16-F10 cells (i.e., B16-LX/RFP) significantly inhibited B16-F10 tumor growth compared to immunization with irradiated B16-F10 cells without virus loading. Importantly, the B16-LX/IL(15 + 7) vaccine demonstrated a significantly superior prophylactic antitumor effect than the control B16-LX/RFP vaccine [31], indicating the antitumor role of dual cytokine expression. Both B16-LX/IL(15 + 7) and B16-LX/RFP vaccines similarly enhanced infiltration of CD4^+^ and CD8^+^ T cells into the TME compared to control irradiated B16-F10 cells. The antitumor response of the B16-LX/IL(15 + 7) vaccine was tumor-specific, as it did not inhibit the growth of antigenically distinct EL-4 lymphomas [31] (Fig. 3; Table 1).

### Viral co-expression of IL-7 plus IL-15 induces tumor-specific and CD8-dependent therapeutic immunity

Like the prophylactic efficacy, the therapeutic B16-LX/IL(15 + 7) vaccine significantly inhibited B16-F10 tumor growth compared to the B16-LX/RFP vaccine, demonstrating the antitumor role of dual cytokines. The antitumor effect of the B16-LX/IL(15 + 7) vaccine was tumor-specific, as the EL4-LX/IL(15 + 7) vaccine (i.e., irradiated EL-4 tumor cells loaded with LX/IL(15 + 7)) did not inhibit antigenically unrelated B16-F10 tumor growth [31]. Mechanistically, although the B16-LX/IL(15 + 7) vaccine significantly increased intratumoral infiltration of CD3^+^, CD4^+^, and CD8^+^ T cells (vs. B16-LX/RFP), its efficacy was abrogated in the absence of CD8^+^ T cells, indicating CD8-dependent efficacy (Fig. 3; Table 1). However, no distinct contributions of IL-7 and IL-15 expression were clearly defined in this study due to the lack of appropriate controls, such as irradiated B16-F10 cells loaded with LX/IL-7 or LX/IL-15 [31]. Thus, future research is necessary to clarify this issue.

## Viral co-expression of IL-7 plus B7.1

B7.1 (CD80) is a glycoprotein receptor recognized as a DC maturation marker [76]. CD80 binds to the CD28 co-stimulatory receptor, activating T cells, or to the CTLA-4 co-inhibitory receptor, downregulating T cell activity [77]. Since IL-7 is involved in T cell activation and maintenance [6, 7, 72] while the CD80-CD28 interaction contributes to T cell activation [77], the viral co-expression of IL-7 and CD80 will likely induce superior antitumor T cell activity. In this context, a replication-defective adenovirus co-expressing IL-7 and B7.1 (Ad.IL-7/B7.1) was developed, and its efficacy was evaluated against transplanted and chemically induced non-transplanted tumors [32], as outlined below.

### Viral co-expression of IL-7 plus B7.1 produces T cell-dependent efficacy against transplanted tumors

Intratumoral injection of a control adenovirus expressing beta-galactosidase (Ad.βgal) inhibited the growth of subcutaneous TS/A adenocarcinoma in BALB/c mice compared to PBS injection. Tumor growth inhibition was more pronounced with intratumoral Ad.IL-7/B7.1 treatment compared to Ad.βgal, leading to 70% (7/10) tumor-free long-term survivors in the Ad.IL-7/B7.1 group, but there were no survivors in the Ad.βgal group (Table 1). This result highlights the antitumor role of IL-7 plus B7.1. However, Ad.IL-7/B7.1 did not show efficacy against established TS/A tumors in BALB/c nu/nu mice which lack T cells, indicating the efficacy of Ad.IL-7/B7.1 is T cell-dependent [32].

Meanwhile, intratumoral Ad.IL-7/B7.1 treatment enhanced the infiltration of CD4^+^ and CD8^+^ T cells into tumors compared to the control. However, it was unclear which T cell subtype (CD4^+^ or CD8^+^ T cells) was primarily responsible for the antitumor efficacy against transplanted tumors. 100% of the tumor-free mice due to Ad.IL-7/B7.1 treatment rejected TS/A tumor rechallenge [32], likely due to the treatment-induced immunological memory (Fig. 2; Table 1).

### Viral co-expression of IL-7 plus B7.1 does not show efficacy against non-transplanted tumors

Although Ad.IL-7/B7.1 was effective against transplanted subcutaneous TS/A adenocarcinoma, intratumoral co-expression of IL-7 plus B7.1 did not show efficacy against 3-methylcholanthrene (3MC)-induced non-transplanted tumors. These non-transplanted tumors were generated by intramuscular injection of the 3MC carcinogen, which typically causes fibrosarcoma-like tumors, or by subcutaneous injection, which generates papilloma-like tumors. The lack of efficacy in this model was likely due to the absence of significant tumor immune infiltrates (CD4^+^ and CD8^+^ T cells) in the Ad.IL-7/B7.1 treatment group compared to the control group [32] (Fig. 2).

Interestingly, in a transplanted fibrosarcoma model derived from 3MC-induced MC51-9 fibrosarcoma cells, intratumoral Ad.IL-7/B7.1 was efficacious, leading to 87.5% (7/8) tumor-free long-term survivors [32] (Fig. 2). The reason for the contrasting efficacy of Ad.IL-7/B7.1 between the two models, i.e., no efficacy against non-transplanted tumors (generated by intramuscular or subcutaneous 3MC injection) versus 87.5% efficacy against transplanted tumors (derived from 3MC-treated MC51-9 fibrosarcoma cells), remains unclear. The authors concluded that the lack of efficacy against non-transplanted tumors was not due to the type or location of the tumor or limited adenoviral gene (IL-7/B7.1) transduction efficiency [32]. Unfortunately, no further studies were reported to understand these differences.

## Viral co-expression of IL-7 and CCL19

While considerable investigations of OVs armed with anticancer cytokines with or without ICIs have been reported [18, 21, 22], another strategy is to encode chemokines that can help attract target immune cells to the tumor site. Indeed, the FDA-approved talimogene laherparepvec (T-VEC) encodes granulocyte-macrophage colony-stimulating factor (GM-CSF), which was designed, in part, to attract local DCs to initiate tumor-associated antigen presentation [25]. Like IL-7, GM-CSF helps in T cell recruitment to the tumors [78]. CCL19, a cytokine that binds to CCR7 (a chemokine receptor), plays a vital role in T cell trafficking [79]. Therefore, CCL19 expression is expected to work synergistically with IL-7 or GM-CSF expression. The positive effects of cytokine expression and immune cell infiltration into tumors may be counterbalanced by the expression of immune checkpoints, such as PD-1 [80]. Consistent with this notion, viral expression of an antibody against PD-1 can reverse PD-1-related T cell exhaustion [81].

A type 2 oHSV was generated to co-express IL-7 and CCL19 (oHSV2-IL7×CCL19) and was then tested with additional type 2 oHSV vectors expressing GM-CSF (oHSV2-GMCSF), IL-12 (oHSV2-IL12), anti-PD-1 (oHSV2-PD1v), and IL-15 (oHSV2-IL15) [82]. The five oHSV constructs demonstrated superior antitumor efficacy, resulting in 100% long-term survivors in two murine tumor models (CT26 and 4T1). The oHSV2-IL7×CCL19 (or oHSV2-GMCSF) treatment showed better efficacy (but statistically insignificant) than the other three viruses (oHSV2-IL12, oHSV2-PD1v, or oHSV2-IL15) in the CT26 model (Table 1) [82]. The specific contribution of IL-7 to the antitumor efficacy, induced either by oHSV2-IL7×CCL19 or a cocktail of five oHSV2s, remains unclear. Further research, including testing the antitumor efficacy of the combination (i.e., oHSV2-IL7×CCL19) versus controls (e.g., oHSV2-IL7, oHSV2-CCL19, oHSV2-IL12, oHSV2-PD1v, or oHSV2-IL15), is needed to define the antitumor role of IL-7 (or other cytokines) expression.

## Conclusions

IL-7 has immunomodulatory properties that can facilitate T cell activation and promote cancer immunotherapy. A major obstacle in driving therapeutic responses by systemic IL-7 cytokine therapy is the narrow therapeutic window exhibited by IL-7. Here, we describe the use of oncolytic and non-oncolytic virus vectors as novel strategies for locally delivering high doses of IL-7 with less systemic toxicity. Various viruses engineered to express IL-7 have now been tested in several preclinical cancer models [27–29], demonstrating superior antitumor immunity via activation of TILs—a hallmark of “immunologically hot” tumors [67]—compared to non-IL7-expressing viruses. The local expression of IL-7 alone, and in combination with other cytokines and chemokines, remodels the TME and enables tumor susceptibility to immunotherapy [29, 67]. In some cases where viral expression of a single cytokine is insufficient to eradicate tumors [27], combination approaches were able to mediate tumor eradication, long-term survival, and the development of tumor-specific immunological memory [45]. Furthermore, viral expression of IL-7 alone, and especially with other cytokines, within tumors improves the antitumor efficacy of CAR-T cell immunotherapy [29], autologous tumor cell vaccines [28], and ICI treatment [30].

While there is strong preclinical evidence that multiple cytokine vectors (e.g., IL-7 plus IL-12, IL-7 plus IL-15, IL-7 plus B7.1, or IL-7 plus CCL19) have superior therapeutic activity in vivo, the absence of appropriate controls in those preclinical reports has been a limitation in understanding the contribution of individual cytokines. Nonetheless, there is intriguing data suggesting that certain IL-7 combinations may be especially interesting. For example, given that IL-12 is a key pro-inflammatory cytokine that promotes T cell proliferation and cytotoxicity [50, 51], while IL-7 supports T cell proliferation, maintenance, and survival [6, 7, 72], the viral co-expression of IL-7 and IL-12 may represent a promising strategy for dual cytokine-expressing viruses in cancer immunotherapy. Recent studies involving an oncolytic vaccinia virus co-expressing IL-7 and IL-12 demonstrated potent antitumor efficacy across melanoma, colon, and lung cancer models [30].

While IL-12 appears to be promising for viral co-expression with IL-7, another cytokine that has not been as well evaluated is IL-2. Like IL-12, IL-2 is another potent proinflammatory cytokine that can also be expressed by viruses [20]. IL-7 synergizes with IL-2, creating an immune-active TME and sensitizing tumors to ICIs [83]. This combination (i.e., IL-7 plus IL-2) could be expressed using replicative oHSVs to induce immune responses with oncolysis [84] or non-replicative oHSVs for sustained cytokine expression and antitumor immunity via a non-lytic mechanism [26]. Tumor models are important, so testing constructs in “immunologically cold” cancer types (e.g., GBM) that are minimally responsive or not responsive to ICIs [85] can be informative. Additionally, it would be worthwhile to virally co-express IL-7 in conjunction with T cell co-stimulatory ligands, such as 4-1BBL [86], OX40L [87], ICOSL [66, 88], or similar ligands [89], which are known to generate potent antitumor immunity. Several oncolytic viruses that have been approved or are under clinical development for cancer treatment express cytokines, including GM-CSF, IFNα, and IL-15 receptor agonists. Further studies of virally encoded IL-7 expression alone and in combination merit further investigation as a strategy for realizing the potential therapeutic value of IL-7 for cancer treatment.
